# Role of Estimated Glomerular Filtration Rate in Clinical Research: The Never-Ending Matter

**DOI:** 10.31083/j.rcm2501001

**Published:** 2024-01-04

**Authors:** Chiara Abenavoli, Michele Provenzano, Sara H. Ksiazek, Lilio Hu, Vania Cuna, Gaetano La Manna, Giorgia Comai, Olga Baraldi

**Affiliations:** ^1^Nephrology, Dialysis and Renal Transplant Unit, IRCCS-Azienda Ospedaliero-Universitaria di Bologna, 40138 Bologna, Italy; ^2^Department of Medical and Surgical Sciences (DIMEC), Alma Mater Studiorum University of Bologna, 40138 Bologna, Italy; ^3^Department of Medicine VI with Nephrology and Dialysis, Clinic Ottakring, 1170 Vienna, Austria

**Keywords:** chronic kidney disease, epidemiology, prognosis, enrichment, endpoint, biomarker

## Abstract

**Background:**

Chronic kidney disease (CKD) burden is crucial both on a global scale and at individual patient level, affecting morbidity and mortality directly and through its effect on both cardiovascular damage and CKD progression to end-stage-kidney-disease (ESKD). Unfortunately, the awareness of CKD is poor, with few CKD patients conscious of the severity of their health status. The principal biomarker of kidney function is estimated glomerular filtration rate (eGFR).

**Methods:**

We searched the literature and present a review article with the aim of summarizing the role of eGFR in clinical research. In particular, we report the eGFR role as a prognostic, enrichment and endpoint biomarker and its role in the early detection of CKD.

**Results:**

eGFR has a major role as a biomarker in clinical research. As a prognostic marker, eGFR reduction is associated with cardiovascular events, ESKD and mortality. As an enrichment biomarker, eGFR values are pivotal for selecting patients to be included in randomized and observational studies; it helps to test a pre-defined drug in early CKD or in more advanced CKD allowing also to avoid screening failures and to shorten the duration of clinical trials. Moreover, eGFR decline (expressed as a percentage of reduction from baseline or continuous slope) can be considered a good endpoint in clinic trials overcoming delays whilst waiting for hard endpoints to develop.

**Conclusions:**

eGFR is a strong clinical measure for both observational and intervention studies. It is also helpful in screening the general population for kidney disease and, in particular, to increase awareness of CKD.

## 1. Introduction

Chronic kidney disease (CKD) is defined as abnormalities of kidney structure or function, present for at least 3 months, with implications for health. Chronic kidney disease is classified based on cause, estimated glomerular filtration rate (eGFR) category (G1–G5), and albuminuria category (A1–A3) [1]. Incidence and prevalence of CKD vary among countries and are mainly influenced by ethnicity and socioeconomic status. The worldwide prevalence of CKD is 13.4% (11.7–15.1%), with thousands of patients (range between 4.902 and 7.083 million) requiring renal replacement therapy (RRT, or also known as end-stage-kidney-disease, ESKD) [2]. Projections from the Global Health Observatory suggest that mortality due to CKD will reach an impressive rate of 14 per 100,000 people by 2030 [3]. The burden of CKD is relevant on a global scale but also at from the perspective of individual patientss, affecting morbidity and mortality directly, and through its effect on both cardiovascular (CV) damage and CKD progression to ESKD. Moreover, the epidemiology trend showed that CKD prevalence and incidence have doubled in the past three decades, increasing by 87% and 89% from 1990 to 2016, respectively [4]. These data gain significance if considering that the awareness of CKD is poor, with only a few CKD patients, less than half, conscious of the severity of their health status [5]. In addition to the epidemiological perspective, it is important to remark that the presence of CKD is, *per se*, associated with an increased risk for CV events, all-cause death and kidney disease progression [6, 7]. All these data are alarming and prompt the need for further effort in the attempt of preventing, or at least relenting, the future trend, and improving individual prognosis. To this aim, the principal strategy that has been advocated is to intensify research in terms of detection of CKD, risk stratification of CKD patients and improving care of these patients [8]. The principal biomarker of kidney function level is represented by the eGFR. The acronym GFR refers to a measure of the sum of the filtration rates of all functioning nephrons, which can be measured or estimated, and which is used in clinical practice to diagnose chronic kidney disease, determine its degree of severity, and establish the prognosis of a patient with CKD, which is also helpful for therapeutic decisions [9]. Similarly, eGFR is widely used in clinical research to select patients to include in clinical trials (enrichment biomarker), to monitor the treatment effect (endpoint biomarker), to predict the progression to ESKD in observational studies (prognostic biomarker). Herein, we present a narrative review, which summarizes the clinical and research contexts in which eGFR is used. With respect to clinical use, we will also present a brief discussion of the central role of eGFR in screening patients with CKD.

## 2. eGFR: Measures and Estimations

The article search for this review was performed in PubMed and was according to author knowledge and experience on the specific field of eGFR. The glomeruli filters approximately 180 liters (L) of plasma per day which corresponds to 125 milliliters/minute (mL/min) of glomerular filtration. Normal GFR values, which are related to age, sex, and body size, are approximately 130 mL/min/1.73 $m^{2}$ in young men and 120 mL/min/1.73 $m^{2}$ in young women [10]. The GFR can be measured through the clearance of exogenous or endogenous markers. An ideal filtration marker must have some key characteristics: it is freely filtered through the glomeruli, neither reabsorbed nor secreted by the renal tubule and is not metabolized. Furthermore, it must not be toxic. Historically, inulin was considered the ideal filtration marker for measuring GFR; however, the measurement of inulin clearance is difficult and invasive, so not practical for daily GFR measurements [11]. Alternatively, urine clearance of iothalamate or plasma clearance of iohexol, both with a great correlation with clearance of inulin, can be used for GFR measurement which also requires the injection of the exogenous marker as well as multiple either urine or blood samples at different times [11]. The measured GFR can be used in usual practice when important decisions require knowledge of the exact level of kidney function, such as the planning of vascular access ahead of dialysis, evaluation of patients with symptoms of uremia, use of contrast media or for kidney donation purposes [12]. As this is often not realistic in daily clinical practice, several equations have been developed over time to estimate (rather than measure) the GFR using plasma levels of endogenous markers such as creatinine and Cystatin C. The blood levels of endogenous markers are however influenced by factors such as the rate of synthesis of the marker, its tubular secretion and/or reabsorption, or the extra-renal elimination. The equations that provide an estimate of the GFR, consider clinical-demographic variables that can modify the GFR itself and are generally acceptable to follow the patient in the clinical practice. The initial approach in evaluating the eGFR is based on creatinine, which is also the most used marker in clinical practice [13]. However, creatinine is not an ideal marker since it can be modified by several factors, including muscle mass, certain medications, as well as diet [14]. Cystatin C is used in clinical practice as a secondary confirmation approach, as it is more reliable in this regard. Studies have shown that Cystatin C is less affected by age, race, diet or muscle mass and may also be used to determine mild changes in eGFR (between 60–90 mL/min/1.73 $m^{2}$) [15]. Nevertheless, although Cystatin C is considered a more reliable marker than creatinine, there is still little information regarding factors that influence this parameter and the costs of measuring Cystatin C are considerably higher [15, 16].

In recent years other biomarkers like β-trace protein (BTP), neutrophil gelatinase-associated lipoprotein (NGAL) or kidney injury molecule-1 (KIM-1) have been discovered as markers for CKD progression but are not yet ready to be widely used in clinical practice and need further investigation [17]. There was a study by Inker *et al*. [18] where an equation using creatinine, Cystatin C, BTP as well as β-2-microglubulin was more accurate than a Cystatin C-based eGFR and as accurate as the eGFR based on creatinine and Cystatin C together however further studies are needed to confirm these findings.

The main equations used to estimate eGFR through creatinine levels are the modification of diet in renal disease (MDRD) and the Chronic Kidney Disease Epidemiology Collaboration (CKD-EPI) equation.

The MDRD equation estimates eGFR adjusted for the body surface, age, gender, serum creatinine and race. The estimation equation is GFR = 186 × (serum creatinine [Scr]) – 1.154 × (age) – 0.203 × 0.742 (if the subject is a woman) or × 1.212 (if the subject is black). The equation was reformulated in 2005 to use a standardized dosage of serum creatinine (lower values of 5%): GFR = 175 × (standardized Sc) – 1.154 × (age) – 0.203 × 0.742 (if the subject is female) or × 1.212 (if the subject is black) [9].

The major limit of the MDRD equation is to underestimate the GFR at higher ranges of kidney function [19]. The 2009 CKD-EPI equation, also based on serum creatinine, was developed with the aim of formulating an equation as accurate as the MDRD at GFR less than 60 mL/min/1.73 $m^{2}$, whilst being more accurate at a higher GFR [20]. The CKD-EPI equation quickly replaced the use of other estimated formulas.

Both the MDRD and the 2009 CKD-EPI place importance on the Black race because previous studies indicated a higher average serum creatinine level for the same measured GFR level in Black participants than in non-Black ones [21]. However, it has been shown that the difference in terms of race has more of a cultural basis than a biological one [22]. From these assumptions, a new CKD-EPI equation was reformulated in 2021 and did not include race in the GFR assessment [23, 24]. The new estimated equation is eGFRcr = 142 × min (Scr/κ, ${\mathrm{1)}}^{\alpha }$× max (Scr/κ, ${\mathrm{1)}}^{-1.200}$× ${\mathrm{0.9938}}^{\text{Age}}$× 1.012 [if female], where κ is 0.7 (females) or 0.9 (males), α is –0.241 (female) or –0.302 (male), min (Scr/κ, 1) is the minimum of Scr/κ or 1.0 and max (Scr/κ, 1) is the maximum of Scr/κ or 1.0. The National Kidney Foundation recommends using the CKD-EPI 2021 equation in clinical practice [25]. Other equations to estimate GFR have been reported but are less used in clinical practice. To estimate clearance of creatinine, the Cockcroft-Gault formula (CG) was created, based on 4 parameters namely serum creatinine, age, body weight and sex. Albeit such an equation has been widely used since its introduction in 1976, it has two main limitations: it is unprecise in obese patients, needing a body weight adjustment and it has been computed by using not standardized creatinine values in the original cohort [26]. However, it should be also highlighted that several drug dose adjustments for kidney function, in clinical practice, are reported using the CG formula. The CKD-EPI also proposed other two equations which include Cystatin C values, namely the CKD-EPI Cystatin (cys) equation and the CKD-EPI Cystatin-creatinine (cys-creat) equation. Although Cystatin C should be used to confirm data based on creatinine, it has been shown that CKD-EPIcys-creat equation is able to correctly reclassify patients being more strictly associated with measured GFR namely the standard of care GFR measurement [27]. The Schwartz-equation, the updated chronic kidney disease in children (CKiD) and the CKiD Under 25 years (CKiDU25) equation were instead developed to estimate eGFR in young/young adult populations [28, 29, 30].

## 3. Association between eGFR and CV Risk

Historically, the association between CKD and increased CV risk has been related to the presence of comorbidities such as hypertension and diabetes, which are per se traditional CV risk factors. Furthermore, the combination of electrolyte abnormalities, anemia, as well as the increase of blood urea are some of the additional factors that contribute to the CV burden in these high- risk patients.

**Fig. 1.**
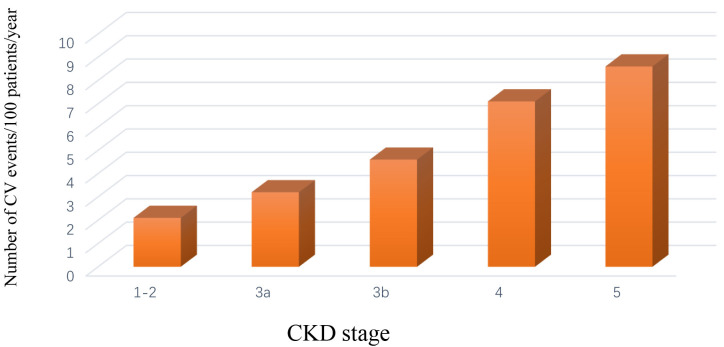
**Rate of fatal and non-fatal cardiovascular events (myocardial infarction, stroke, heart failure, peripheral vascular disease) in chronic kidney disease patients stratified by stage [32]**.

However, several studies have demonstrated that eGFR acts as a strong predictor of CV events (mainly coronary heart disease, chronic heart failure, stroke, peripheral vascular diseases, CV death), regardless of the presence of any other comorbidities and clinical or demographic variables such as age or gender. Moreover, such an association is present in patients with already assessed CKD, namely those already under the care of a nephrologist, but also in subjects derived from the general population [6]. Large studies including CKD patients showed that considering 100 mL/min as a reference point for a low eGFR , the risk of CV fatal and non-fatal events was almost doubled [31]. In a cohort of CKD patients followed up by nephrologists in 40 Italian centers, the incident rate of fatal and non-fatal events over time was progressively higher moving from CKD stage 1–2 to 5, with a relative risk of 48% moving from one stage to the next more severe stage (Fig. 1) [32].

In a recent meta-analysis which enrolled around 10,000 individuals from the general population, the independent association between eGFR reduction and future CV events, regardless of previous history of CV disease was demonstrated. Particularly, a higher risk of CV death is evident in patients with eGFR level ≤60 mL/min/1.73 $m^{2}$ compared to people with normal kidney function, with this risk being two-fold higher with a eGFR value of 30-45 mL/min/1.73 $m^{2}$ [33]. CV risk associated with CKD is also independent of the presence of diabetes and hypertension as well [34].

From a prognostic perspective, the current risk scores including traditional risk factors only (age, blood pressure, low density lipoprotein (LDL) cholesterol, smoking habit and gender) underestimate CV risk in CKD cohorts [35]. Overall, the inclusion of the two “kidney measures”, eGFR and albuminuria, significantly improve CV risk prediction both in the general population and in high risk patients such as CKD patients [35]. Besides the traditional risk factors, inactivity may also be an important predictor of mortality as there have been studies linking CKD with low exercise levels as well as improved hazard ratios in patients who did exercise [36, 37]. The Gruppo di Lavoro Italiano Sarcopenia-Trattamento E Nutrizione (GLISTEN) study highlighted the connection between eGFR and mortality in older patients, with the highest being in patients with an eGFR <35.32 mL/min/1.73 $m^{2}$ and a Short Portable Status Mental Questionaire (SPMSQ) ≥5 (hazard ratio (HR): 5.49, 95% confidence interval (CI): 3.04–9.94) [38].

Unfortunately, the exact mechanism by which eGFR decline increases risk of CV risk is only partially understood.

Several factors may play a crucial role. For instance, the imbalances in matrix metalloproteinases (MMPs) in CKD patients have been associated with profibrotic and pro-inflammatory mechanisms with subsequent structural changes that lead to atherosclerotic plaque maturation along with arterial remodeling [39]. This results in an increased risk of arterial wall pathologies in CKD patients such as aneurism complications and atherosclerotic disease. Furthermore, the eGFR reduction in CKD patients is associated with a persistent, low-grade inflammation which correlates with increased CV risk. Different factors such as the decreased elimination of cytokines, metabolic acidosis, as well as the oxidative stress and the recurrence of infections contribute to the inflammatory status that characterizes CKD patients [40]. Moreover, studies have shown that these changes contribute to the development of heart failure with preserved ejection fraction (HFpEF) in patients with CKD [41]. It was also possible to detect a specific set of biomarkers which predict all-cause and CV mortality with more accuracy than other biomarkers: increased values of interleukine (IL)-6 are a stronger predictor than other cytokines such as tumor necrosis factor (TNF)-α, IL-1β and IL-18 [42]. The link between eGFR and CV risk has been confirmed in autopsy studies that showed how the grade of kidney impairment correlated with the severity of coronary atherosclerosis, even in patients without previous CV disease. A Japanese cross-sectional study, in which 126 individuals were randomly selected from 844 autopsy samples, showed that advanced atherosclerotic lesions were inversely correlated with eGFR value: their frequency increased as eGFR decreased (33.6%, 41.7%, 52.3%, and 52.8% for eGFRs > or = 60, 45–59, 30–44, and <30 mL/min/1.73 $m^{2}$, respectively; *p* for trend = 0.006). Even calcified lesions of coronary arteries rose gradually with lower eGFR values (*p* for trend = 0.02) [43]. Other studies have also highlighted the relationship between CKD and coronary artery disease as well as with atrial fibrillation (AF), with CKD being an independent risk factor of AF [44, 45, 46].

Further investigations are needed to better understand the link between eGFR decline and the increased CV risk.

## 4. CV and CKD Progression Risk Reduction Associated with Novel Nephroprotective Treatments 

The evaluation of kidney function is also important from an interventional perspective, namely when considering eGFR decline as a modifiable (by therapies) risk factor. Different interventional studies such as those aiming to reduce blood pressure levels have shown benefits in terms of both renal and CV risk due to a slower rate of eGFR decline. This is the case with renin-angiotensin-aldosterone-system inhibitors (RAAS-I): these drugs warranted a reduction in relative risk of CV events of 22% in the ramipril-treated group, compared to the placebo-treated group, in the heart outcomes protection evaluation (HOPE) study [47].

In the past few decades, new promising drugs have been developed and approved for CV risk reduction in CKD patients, particularly the sodium-glucose cotransporter 2 inhibitors (SGLT2-is) and novel non-steroidal mineralocorticoid receptor antagonists (MRAs). The SGLT2-is act by reducing the reabsorption of glucose in the renal proximal tubule [48, 49, 50]. The Canagliflozin Cardiovascular Assessment Study (CANVAS) reported the significant benefit of the SGLT2-i canagliflozin, in reducing the composite outcome (CV death, nonfatal myocardial infarction or stroke) by about 15% in patients suffering from type 2 diabetes (DM2) with elevated CV risk both for primary and secondary prevention [51].

Another paramount study is the EMPA-REG OUTCOME trial (Empagliflozin Cardiovascular Outcome Event Trial in Type 2 Diabetes Mellitus Patients) where SGLT2-i empagliflozin was shown to reduce CV morbidity and mortality in DM2 patients with eGFR ≥30 mL/min. In patients with worse kidney impairment at the baseline, empagliflozin reduced risk for CV death, all-cause mortality and hospitalization for heart failure by 29%, 24% and 39%, respectively [52].

A recent meta-analysis of the Empagliflozin Outcome Trial in Patients With Chronic Heart Failure With Reduced Ejection Fraction (EMPEROR)-reduced and the Dapagliflozin and Prevention of Adverse Outcomes in Heart Failure (DAPA-HF) trials showed that SGLT2-i empagliflozin and dapagliflozin respectively, are associated with a 14% reduction in CV death in patients with reduced ejection fraction with or without diabetes (pooled HR: 0.86, 95% CI: 0.76–0.98; *p = *0.027). Moreover, SGLT2-i treatment was associated with a 26% relative risk reduction of the combined outcome of CV death or first hospitalization for heart failure, and with a 25% decline of the composite outcome of recurrent hospitalizations for heart failure or CV death. The risk of the composite renal endpoint was also reduced (HR: 0.62, 95% CI: 0.43–0.90; *p = *0.013) [53].

The novel MRAs have also been shown to improve CV prognosis in CKD patients. Mineralocorticoid receptor (MR) is overactivated in both CKD and heat failure (HF) with a subsequent increased expression of inflammation and fibrotic pathways, which lead to organ injury [54]. As a protagonist of two important studies, Finerenone in Reducing Kidney Failure and Disease Progression in Diabetic Kidney Disease (FIDELIO) and Finerenone in Reducing Cardiovascular Mortality and Morbidity in Diabetic Kidney Disease (FIGARO), as well as their combined analysis (FIDELITY), the novel MRA finerenone has shown, in patients with CKD and DM2, not only to improve renal function but also to reduce CV risk, with lower incidence of hyperkalemia, as compared to steroidal MRA, albeit confirming pro-inflammatory and profibrogenic pathways [55, 56, 57]. The Finerenone in Reducing Kidney Failure and Disease Progression in Diabetic Kidney Disease (FIDELIO-DKD) double-blind trial, which included 5734 patients with both DM2 and CKD, showed a significant effect of finerenone in reducing the progression of CKD and CV event rate [58]. Moreover, in the Finerenone in Reducing Cardiovascular Mortality and Morbidity in Diabetic Kidney Disease (FIGARO-DKD) trial, treatment with finerenone, compared to placebo, was shown to reduce new-onset HF (1.9% versus 2.8%; HR: 0.68, 95% CI: 0.50–0.93; *p = *0.0162) and to ameliorate HF outcomes in patients with DM2 and CKD, regardless a previous history of HF [59]. The crucial point of discussion is that the CV risk reduction in almost all these studies was present, especially in patients who showed a lower eGFR decline after the commencement of treatment.

## 5. Estimated GFR as Prognostic Biomarker

**Fig. 2.**
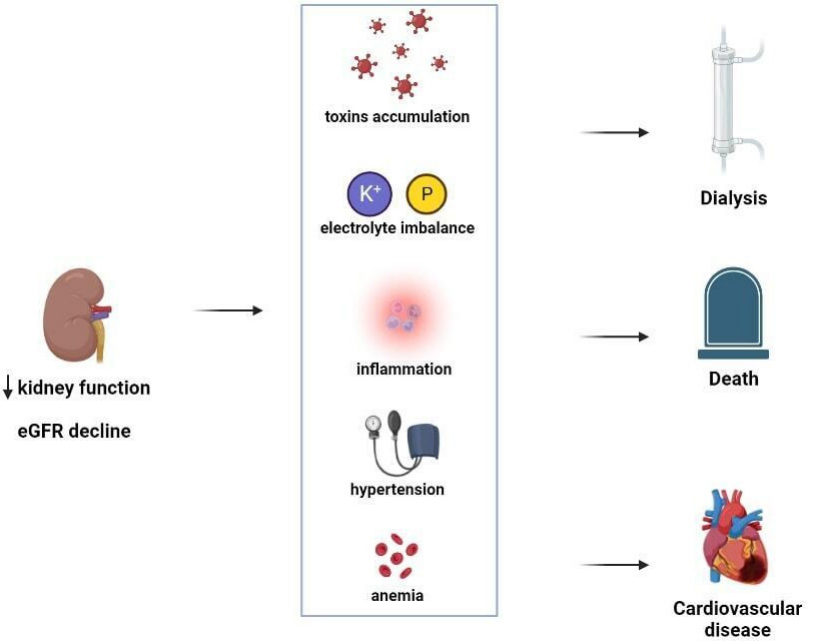
**Estimated estimated glomerular filtration rate (eGFR) and its association with several pathophysiologic mechanisms**. Estimated GFR reduction is associated with the development of several comorbidities (increase in blood toxin levels, electrolyte imbalances such hyperkalaemia and hyposodiemia, inflammation, arterial hypertension and anemia) that, taken together, dramatically increase the risk for future events.

Besides the association of eGFR with CV risk previously discussed, this marker also has a pivotal role in predicting other important outcomes such as CKD progression and all-cause mortality [6]. The association between increased mortality risk and reduced renal function may also in part be explained by the inflammatory milieu of CKD, characterized by oxidative stress, vascular damage and endothelial dysfunction which lead to an increase in global mortality risk and progression of kidney damage [60, 61]. An even more interesting finding is the association between increased eGFR variability and mortality rate, especially when compared to a stable kidney function. In this regard, Turin* et al*. [62] demonstrated that mortality rates were highest for people with an increase in eGFR of 5 mL/min/1.73 $m^{2}$ per year or more and for those with a decline in eGFR of less than or equal to 5 mL/min/1.73 $m^{2}$ per year. Similar results were obtained for increasing and declining percentage changes in eGFR [62]. The CKD Prognosis Consortium represents one of the major studies of CKD populations that highlights the importance of both declining eGFR , together with the increase in albuminuria, as independent factors correlating with ESKD and mortality [31]. All the analyzed studies showed a positive association between a reduction in eGFR and mortality. In particular, 7 out of 8 studies revealed a significant HR for eGFR values of 15–29 mL/min/1.73 $m^{2}$ compared to 45-74 mL/min/1.73 $m^{2}$. Analysis of studies related to the association between eGFR reduction and progression to ESKD showed that of the 11 studies analyzed, 9 had a significantly higher HR for an eGFR of 30–44 mL/min/1.73 $m^{2}$ compared to 45–74 mL/min/1.73 $m^{2}$, while all 11 studies had a substantially elevated HR for an eGFR of 15–29 mL/min/1.73 $m^{2}$ compared to 45–74 mL/min/1.73 $m^{2}$. The association between eGFR reduction and poor prognosis in terms of mortality and renal risk was shown in the general population, in high-risk patients such as individuals with hypertension and diabetes as well as in CKD patients already referred to nephrologists and who are for consequently better treated to prevent future events [63]. A meta-analysis conducted on more than 100,000 subjects from the general population whose albumin-to-creatinine ratio (ACR) values were available, showed that both eGFR and albuminuria values are associated with all-cause death and mortality independently of each other and from other risk factors. An exponential increase in the risk of death from low eGFR levels was observed. The risk becomes statistically significant from eGFR values of approximately 60 mL/min/1.73 $m^{2}$ and becomes 2-fold greater for eGFR values of approximately 30–45 mL/min/1.73 $m^{2}$ when compared to optimal eGFR levels, regardless of the values of albuminuria. These results refute the notion that mild or moderate reductions in eGFR are not associated with adverse clinical consequences [6]. Moreover, an annual decline in eGFR of more than 3 mL/min/1.73 $m^{2}$ has been associated with an increased risk of all-cause and mortality, compared with a reduction of less than 3 mL/min/1.73 $m^{2}$, even after adjustment for confounders [64]. Similarly, a decline of at least 20% in eGFR over an 18 month period has been demonstrated to be predictive of a 1.5 fold higher risk of all-cause mortality (adjusted HR: 1.45; 95% CI: 1.13–1.86) at 15 years in comparison with any decline in eGFR [65]. The reasons underlying these negative associations are multiple and only partially explained. From a pathophysiological perspective, low eGFR means an increase in the burden of uremic toxins, inflammation and pro-atherosclerotic factors such as imbalances in matrix metalloproteinase expression (Fig. 2) [66]. Autopsy studies in patients without traditional risk factors of CKD have shown that the severity of CKD itself is responsible for a sensitive increase in vascular atherosclerotic damage [67]. A certain amount of cardiorenal risk is related to the comorbidities of CKD which normally appear as eGFR declines such as hyperkalemia, hyperparathyroidism, increased serum phosphate levels, dyslipidemia, metabolic acidosis and hyperuricemia [68].

## 6. eGFR as Enrichment Biomarker

The selection of patients to be included in randomized studies is a challenging topic since clinical trials with novel drugs may in one sense reflect the clinical practice world and be applicable in such direction. Normally, one strategy to select patient is based on the levels of certain biomarkers, the so-called biomarker enrichment [67]. Levels of kidney function are detrimental to select patients to be included in randomized studies. The first big trials demonstrating the protective effect of RAAS inhibitors on CKD progression, such as the Reduction of Endpoints in NIDDM (non-insulin-dependent diabetes mellitus) with the Angiotensin II Antagonist Losartan (RENAAL) and Irbesartan Diabetic Nephropathy Trial (IDNT) studies, used serum creatinine as enrichment biomarker [68]. Subsequently, creatinine has been replaced by eGFR levels to the same aim, which may help to account for differences in gender, as the eGFR formulas have a correction factor for gender. Moreover, when using eGFR, a pre-defined drug can be tested in early CKD (e.g., eGFR >60 mL/min with albuminuria) or in more advanced CKD (e.g., eGFR 30–60 mL/min). One further relevant step forward was the discovery as well as application in practice that a large range of eGFR may help to avoid screening failures and to shorten the duration of the clinical trial [69, 70]. Furthermore, one recent hypothesis that is gaining momentum is that the eGFR slope (trajectory over time based on at least three eGFR measurements) may represent a proper inclusion criterion for randomized studies. Based on a post-hoc analysis of the Study of diabetic Nephropathy with Atrasentan (SONAR) trial, it was identified that the treatment effect of nephron-protective therapies may depend on the rapidity of CKD progression over time [71]. These important findings are going to influence the design of future trials. The PRIME-CKD study (https://cordis.europa.eu/project/id/101095146), which will test the individual response of CKD patients to drugs with different mechanisms of action, reports among inclusion criteria a pre-trial slope of at least 1 mL/min per year. Owing these evidence, we can assert that eGFR has a main role as enrichment biomarker in clinical trials and that further studies may reveal how to use this measure in the most appropriate way.

## 7. eGFR as Endpoint Biomarker

An endpoint of a specific study is a measure (also called event) that registers a significant change in the quality of life or, in most negative and unfortunate scenarios, the end of life. A sufficient number of endpoints is mandatory to have a good study power and answer the study question appropriately, in both observational and interventional studies. In this context, eGFR is considered a measure of kidney function decline over time and therefore works as an endpoint, modeled in different mathematic forms. In observational studies, using eGFR as an endpoint is commonly reported as the percentage of eGFR decline from baseline evaluation (e.g., 30%, 40%, 57% eGFR decline) or continuous eGFR slope. In both cases, eGFR endpoints are a surrogate of another major end-point, namely ESKD. The advantage of using eGFR decline as an endpoint is the fact that hard endpoints such as mortality, CV events and ESKD need many years, often decades, to develop. Hence, having more events in a brief period of a certain endpoint which can be considered a surrogate of these other major events is instrumental in clinical research. Several previous studies showed that reductions of less than 57% in eGFR values over time can be used as alternative endpoints for evaluating the progression of CKD. A study conducted on individual meta-analysis data of 1.7 million participants from 35 cohorts in the Chronic Renal Failure Prognosis Consortium evaluated the possibility of using less than 57% reductions in eGFR values over a period of two years to predict the development of ESKD [72]. Furthermore, since many CKD patients die before reaching ESKD, the associated mortality risk has also been assessed. The hazard ratios of ESKD and mortality were higher for larger reductions in eGFR. The 10-year mean risk of ESKD was also calculated in patients with a baseline eGFR value of 35 mL/min/1.73 $m^{2}$ resulting in 99% for 57% reductions in eGFR, 83% for a reduction of 40% in eGFR values, 64% for decreasing of 30% in eGFR and 18% for constant eGFR values. The corresponding mortality risk was 77%, 60%, 50% and 32% respectively, showing a similar but weaker trend. This study showed that a 30% reduction in eGFR values over a two-year period is associated with a 5 times greater risk of ESKD and a 2 times greater risk of mortality [72]. Furthermore, it is important to consider that various risk factors play a fundamental role in the progression of CKD, some of which are time-dependent. A study conducted on 701 patients with CKD has shown how a reduction of 30% in eGFR over a two-year period is associated with the highest HR values of 31.6 for ESKD, whereas the addition of baseline values of eGFR, proteinuria, serum albumin and haemoglobin led to a more accurate prediction model [73]. Nowadays, many observational studies report a prognostic model using an eGFR reduction of 30 or 40% as an endpoint combined with ESKD [73, 74]. The eGFR annual slope, with at least three measures over time, is also frequently predicted as the endpoint in observational studies [75]. This measure is extremely helpful to evaluate the prognosis of CKD patients in the first years of observation. A problem related to the study based on the eGFR slope is also represented by the heterogeneity of the data available for individual patients, which could affect the interpretation and validity of the results. To homogenize these data, a useful approach is the analysis of the slope of the eGFR over time delivering a potential alternative in terms of endpoints in clinical studies.

Several landmark studies in the field of nephrology from recent years have used both the percentage eGFR reduction as well as continuous eGFR slope to demonstrate the response to nephron-protective therapies. In a detailed analysis of the EMPA-REG-OUTCOME trial, the presented eGFR slope showed that empagliflozin has the potential to slow the decline of eGFR after treatment for around three years, even in patients at higher risk of worsening CKD [76]. Another work that has supported the eGFR slope as a possible future endpoint in chronic kidney disease progression studies is a study conducted in patients with DM2 being treated with Canagliflozin [77]. Most of the novel trials, testing the efficacy of endothelin receptor antagonists, mineralocorticoid receptor antagonists or SGLT2 inhibitors reported the analysis of treatment effect on eGFR slope [72, 78, 79]. The percentage of eGFR decline has been also evaluated as an outcome of interventional studies. 40% and 57% eGFR reduction from baseline (start of treatment visit) are considered good endpoints of response to treatment, even in clinical scenarios of a rapid, yet only functional, eGFR decline immediately after the treatment initiation [71, 79]. Interestingly, data from the FIGARO-DKD study suggests caution in interpreting the 40% eGFR decline. In fact, the effect of finerenone in this trial was significant when tested on 57% eGFR reduction and not significant (borderline confidence interval) on 40% eGFR decline suggesting that potentially in some occasions, more powerful (and not more frequent!) events may reveal treatment effects more clearly. These alternative endpoints are also useful in randomized studies for enrolling patients at early stages of kidney disease, where hard endpoints occur after many years and therefore surrogate endpoints are needed. On the other hand, testing the efficacy of nephroprotection at an early stage of CKD is the main aim of clinical research. The role of eGFR for clinical and research purposes has been already developed in several review articles as reported in Table 1 [9, 80, 81, 82, 83, 84, 85, 86, 87], whereas the main contents and novelties of the present review are repored in Table 2 (Ref. [6, 60, 62, 63, 64, 68]).

**Table 1.** **Principal review articles describing the role of estimated glomerular filtration rate**.

Article	First author	Main concept
Assessment of Glomerular Filtration Rate in Health and Disease: A State of the Art Review [80]	A.S. Levey & L.A. Inker	GFR is the main index of kidney function. It has a paramount role in the kidney disease care and in drug dosing. GFR should be estimated using the most accurate GFR estimating equation, particularly the CKD-EPI based on creatinine or Cystatin C.
Evaluating the Performance of GFR Estimating Equations [81]	L. A. Stevens	Estimated GFR represent a valid tool in the management of CKD. Despite the limitations related to the lack of a single eGFR equation that applies to all people, eGFR equations are crucial in clinical research and healthy policy related to CKD.
GFR Estimation: From Physiology to Public Health [82]	A.S. Levey	eGFR equations are less subjected to risk of bias when used in CKD patients compared to the general population. Further improvement in eGFR equations are needed in order to better represent differences in populations, to use multiple filtration markers, and to use statistical techniques to compare eGFR to measured GFR.
Measured and estimated glomerular filtration rate: current status and future directions [83]	A.S. Levey	GFR is considered the main marker of kidney function. Estimated GFR (eGFR) and measured GFR (mGFR) are both associated with errors compared to the actual GFR (measured GFR, mGFR). While further adjustment of the GFR calculation equations is required, eGFR is recommended for initial filtrate assessment, with mGFR generally considered an important confirmation test.
Measurement and Estimation of GFR for Use in Clinical Practice: Core Curriculum 2021 [9]	L.A. Inker & S. Titan	The GFR is used in clinical practice and research as the main tool for diagnosing, staging and managing CKD as well as for defining CKD-related prognosis and mortality risk. GFR is estimated using equations using the serum creatinine level. However accurate, GFR estimation may require confirmatory tests of which currently available include equations based on Cystatin C, urinary or plasma clearance of markers of exogenous filtration or urinary creatinine clearance.
New and old GFR equations: a European perspective [84]	P. Delanaye	GFR is considered a cornerstone tool in CKD. However, all the equations available for calculating GFR give a rough estimate rather than the actual value. A precise measurement of GFR in specific populations and/or specific clinical situations might be required.
Determining the Glomerular Filtration Rate—An Overview [85]	E. Schaeffner	The GFR determination is crucial in clinical practice as it helps, among other things, in therapeutic decisions. GFR can be calculated or more commonly estimated using several formulas. The most used filtration marker to date is creatinine, although recently Cystatin C is taking on an increasingly important role. Furthermore, new formulas for GFR evaluation, applicable to all ages, are emerging.
Estimated Glomerular Filtration Rate in Chronic Kidney Disease: A Critical Review of Estimate-Based Predictions of Individual Outcomes in Kidney Disease [86]	L. Zsom	GFR assessment is a useful tool in clinical practice being also used as a prognostic indicator of chronic kidney disease progression. However, it should be considered as an initial screening tool, since its reduction over time should be evaluated overall together with the general clinical context.
Estimating glomerular filtration rate: is it good enough? And is it time to move on? [87]	D. P. Murphy	The evaluation of GFR is fundamental in daily clinical practice. Several studies have demonstrated the non-superiority of GFR measurement compared to its estimation using formulas in predicting renal outcome.

(e)GFR, (estimated) glomerular filtration rate; CKD, chronic kidney disease; CKD-EPI, Chronic Kidney Disease Epidemiology Collaboration.

**Table 2.** **Summary of the main concepts derived from this study**.

eGFR as	Key-concepts
Prognostic biomarker	- The eGFR variability is associated with higher risk of all-cause death and CV events [62].
	- Estimated GFR decline is a strong predictor of CKD progression (particularly ESKD), CV events and mortality [60].
	- Even mild or moderate reductions in eGFR have potentially important clinical sequelae [6].
	- The cardiorenal risk related to eGFR declines is impaired by the presence of CKD comorbities such as hyperkalemia, hyperparathyroidism, increased serum phosphate levels, dyslipidemia, metabolic acidosis and hyperuricemia [68].
Enrichment biomarker	- Kidney function values are detrimental to select patients to be included in randomized and observaitonal studies [64].
	- The evaluation of eGFR values not only allows a pre-defined drug to be tested in early CKD or in more advanced CKD but also it helps to avoid screening failures and to shorten the clinical trials duration [63].
	- The eGFR trajectory before study initiation (pre-trial eGFR slope) may inform about the treatment effect and thus it can be considered an important enrichment criterion in future research.
Endpoint biomarker	- Estimated GFR decline (expressed as percentage of reduction from baseline or continuous slope) can be considered a good endpoint in clinic trials.
	- The advantage of using eGFR decline as endpoint is the fact that the hard endpoints need many years, often decades, to develop.
	- The continuous eGFR slope is considered an accurate measure of eGFR changes over time since it encompasses severel eGFR values over time and it also may account for the presence of other confounding variables.

(e)GFR, (estimated) glomerular filtration rate; CKD, chronic kidney disease; ESKD, end-stage-kidney-disease; CV, cardiovascular.

## 8. Awareness of CKD: The Pivotal Role of eGFR

More than 850 million people worldwide have CKD and by 2040 CKD is predicted to be the fifth most prevalent chronic condition in the world [88, 89]. Although the number of individuals affected by CKD is so high, only one in three patients with CKD get diagnosed [90]. This can be attributed to the fact that CKD, especially in early stages is a silent disease, meaning most patients remain asymptomatic until the disease progresses to advanced stages [91]. Another important factor is that there is no standardized systematic screening and treatment strategy for CKD. Moreover, CKD is often viewed as a complication of diabetes mellitus or hypertension but not as a disease itself. As all of this leads to CKD being underdiagnosed, the referral to a nephrologist often does not happen until it is too late and CKD advances to the later stages, where the initiation of dialysis remains inevitable [92]. Further prompting the need to diagnose CKD earlier and prevent disease progression is the established fact that dialysis, although improvement has been made in the last decades, is still associated with incredibly high mortality rates of 10 to 20 times greater than the general population and consumes 5–7% of total health care budgets [86].

The Kidney Disease: Improving Global Outcomes (KDIGO) guidelines define CKD as a decreased eGFR (<60 mL/min per 1.73 $m^{2}$) and albuminuria for a duration of at least 3 months [1]. However, the most common first pathological finding leading to a CKD diagnosis is solely a decreased eGFR. In most middle- to high-income countries, the eGFR gets calculated automatically when ordering a creatinine blood test and therefore already easily provides the means for the screening of CKD [93]. It must be noted though, that the use of the CKD-EPI eGFR formulation is being recommended by the KDIGO guidelines 2012 but some laboratories may still use MDRD for eGFR calculation [94]. The KDIGO controversies conference 2021 also states that CKD diagnosis must consist of a dual assessment of eGFR and albuminuria and that an accurate GFR estimation includes the use of both creatinine and Cystatin C measurement, if the latter is available in middle- to high-income countries [95]. Especially in low-income countries where ACR is not affordable, urine dipstick tests as well as calculation of ACR from protein-to-creatinine ratio PCR measurements can be used as a less accurate alternative to ACR measurement [96].

According to the WHO, principles for screening of a disease include the disease being an important public health problem affecting a large group of individuals, availability of a suitable test or examination as well as treatment options [97]. All those criteria are met with CKD as it is even sometimes described as a global epidemic, eGFR and albuminuria testing is cost effective and treatment options are available [98, 99, 100]. Especially in the last couple of years, with the results from large scale studies showing that SGLT2 inhibitors or nonsteroidal mineralocorticoid receptor antagonists (nsMRA) additional to the already established angiotensin-converting enzyme inhibitors (ACEi) and sartans can reduce mortality and slow progression of CKD, the focus in nephrology has shifted from replacing the kidney via dialysis to actually preventing ESKD [99].

In order to fulfill this goal and to increase the number of patients diagnosed with CKD, the awareness of CKD as a global burden has to rise [100]. The first question that must be answered in this context is, which individuals should be screened for CKD. The KDIGO guidelines on CKD from 2012 omit the screening for CKD completely, which is why in 2021 a KDIGO controversies conference was held to help answer that question [90]. In this conference, risk groups were defined which should be screened by general practitioners or doctors from other specialities, as screening for CKD seldom happens by a nephrologist. The defined risk groups primarily included patients with diabetes, hypertension, or CV disease as well as patients with obesity, family history of renal disease, acute kidney injury (AKI) in patient history, older age, and other high-risk comorbidities. As stated above, in those individuals an assessment of the glomerular filtration by eGFR calculation as well as an assessment of the kidney injury by ACR measurement should be performed. The eGFR together with the ACR are used to stage the patient according to the KDIGO heat map from the 2012 guidelines [69]. This staging allows for risk stratification which is crucial to determine the prognosis of CKD as well as adopt therapy. Another important aspect of risk stratification is to determine high-risk patients who should be referred to a nephrologist [90]. A tool that can be used is the kidney failure risk equation (KFRE) by Tangri *et al*. [100] which provides a 2- and 5-year risk of ESKD by using four variables: eGFR, sex, age and albuminuria. The KFRE was developed in the Canadian population but has been externally validated in 31 multinational cohorts and is therefore applicable worldwide [101]. In the KDIGO controversies conference the use of risk equations like the KFRE is encouraged to stratify CKD patients [90]. The National Institute for Health and Care Excellence (NICE) guidelines 2021 on CKD diagnosis and management even defined a 5-year risk of having ESKD >5% calculated by the KFRE as a referral criterion to a nephrologist and the new KDIGO CKD guidelines, which are in public reviewing by the time of writing this article use a 5-year ESKD risk of 3–5% as referral criteria [1, 102].

How often a patient should be screened remains an individual decision, based on the risk stratification and can range from 1 to 10 years [90].

In order to achieve a comprehensive early identification of CKD and lessen the global burden awareness of CKD has to rise. The new KDIGO CKD guidelines, which are to be published in 2023, finally provide a chapter on CKD screening, but further measures must be undertaken on a global and national level [1]. This most importantly includes education of general practitioners and enabling joint efforts of nephrologists, general practitioners as well as doctors from other specialities to identify and treat CKD.

## 9. Conclusions

To our knowledge, this is the first manuscript that reports a discussion about eGFR in agreement with the standard of clinical research tools, namely its role as a prognostic biomarker, treatment response predictive biomarker and endpoint biomarker itself. Considered together, these points offer a uniform and complete discussion on the topic and better integrate with future perspectives. Moreover, we also reported, in keeping with the need to continue working on this, the role of eGFR in the awareness of CKD, which is gaining momentum among nephrologists and the public health community. We may contend that eGFR is a strong clinical measure for nephrologists and physicians. It significantly helps to refine risk stratification of patients, to include patients in clinical studies and to assess the response to nephro- and cardioprotective treatments. Moreover, eGFR is a useful and very cheap tool, combined with urine examinations, to screen the general population for kidney disease and, thus, to increase awareness of CKD overall. Future studies are needed to implement the use of eGFR in clinical research and practice.
